# What Kills Patients after Laparatomy—Anesthetic, Nephritis, or Infection?

**Published:** 1893-12

**Authors:** F. Byron Robinson

**Affiliations:** 34 Washington Street; Chicago, Ill.


### WHAT KILLS PATIENTS AFTER LAPARATOMY—ANESTHETIC, NEPHRITIS, OR INFECTION?

By F. BYRON ROBINSON, Chicago, Ill.

For some time I have watched the effect of the anesthetic, kidney trouble, and infection following abdominal sections. The subject well repays careful study. In 1885, I gave ether to a perfectly healthy girl of about twenty years of age for one hour and a half. No trace of previous ill-health could be found in her life. The ether was given to extract sixteen teeth. Immediately after the administration of the ether,—*i. e.*, a few weeks, she was taken ill. I found that she had nephritis and albumin in the urine, which increased for some months, and lasted almost a full year. I noticed that it followed the anesthetic, and since have observed nephritis in other cases following anesthetics and laparatomy. Now, it seems to me that there can be but little doubt that nephritis is a frequent sequel to ether administrations. The nephritis follows a few months after, or, rather, it is detected a few months after by examining the urine and finding albumin in it. Ether affects the kidney, occasionally, detrimentally. But this may be on account of the fact that the kidney is already considerably diseased, and that the anesthetic simply precipitates matters. It may excite the old kidney disease, or irritate the remaining portion of the kidney intact. At any rate, ether anesthesia is liable to induce disease of the kidney. Chloroform is so effective on the heart, and I have had so many narrow escapes from death that I almost entirely discard chloroform in laparatomy. In England, either the patient or the chloroform seem to act safer, for in large numbers of anesthesias I never saw any trouble, but it does not act so safe in this country.

The next subject is *nephritis* after laparatomy. Few laparatomists seem to give much attention to the kidney in their work. Many neglect to prepare the kidney for the ordeal of the operation. But one thing is certain: that abdominal tumors soon induce diseases of the kidney. Doran found in autopsies of women who died from ovarian tumors, or immediately after laparatomy to remove the tumor, that the kidney presented morbid symptoms in some eighty per cent. of the cases. Eighty per cent. of complicating disease is a factor too large to be accidental. It is common, in my own practice, in observing women with tumors, to see kidney disease accompanying them. In most cases the urine is scant and high colored, with pink urates. There is a frequent variation in the quantity. Occasionally the urine will be profuse in quantity and clear; but generally scant urine of high color is voided by women with tumors. Again, if one will take pains to examine the urine of women possessing an abdominal tumor, he will find that the urea varies very much.

In a series of cases of laparatomy at the Woman's Hospital of Chicago, I had the urine carefully examined for urea, and it was found varying from four to eleven grains to the ounce. After lying in bed for a few days, the urea would average about seven grains to the ounce—an amount which I consider safe for abdominal section. Kidney disease coëxisting with abdominal tumors is no accident or incident. It is a pathological factor of importance. I have noted it for some time; but it must not be considered that the kidneys alone suffer from abdominal tumors. Every abdominal and thoracic organ is more or less a sufferer. The heart first becomes hypertrophied and then ends in a degree of fatty degeneration. The liver suffers hypertrophy and fatty degeneration. The causes of visceral derangement from abdominal tumors is *reflex*. Pressure, producing chiefly obstruction of ducts, is another factor of less activity. The irritation produced by the tumor is carried to the abdominal brain by the hypogastric and ovarian plexus, or any adjacent flexes which may be irritated. In the abdominal brain the irritation is re-organized and emitted to every viscus, and especially severely emitted to viscera containing large nerve flexes. So far as I can make out in carefully dissected bodies, the kidney is supplied with the largest nerve flexes of any organ, except the uterus; hence, the effect of reflex irritation on the kidney from a tumor is very severe. The reflexes go on night and day, Summer and Winter, with irregular constancy. The effect on the kidney may be stated to be of three kinds: (*a*) The secretion of urine is made excessive; (*b*) it is made deficient, and (*c*) it is made disproportionate. Disproportional secretion is the most disastrous. If the kidney is continually irritated, and, consequently, its secretions unbalanced, it will finally become diseased in structure. It will end in tubular nephritis or interstitial nephritis. If pressure of the ureters plays the *rôle*, obstruction of urine follows. Obstruction, reflex irritation, and congestion is frequently followed up by infection in the kidney, the disease travels up the ureter, the kidney pelvis becomes dilated, the kidney atrophies. Uterine myomas are very productive of kidney disease, no doubt, because they produce much friction, irritation, and reflex action. It may be observed that the connection of the kidney to the genital organs is very intimate. The kidney and genitals all developed out of the Woelfian body; hence, they are connected by nerves and vessels. Disturbance in the genitals soon disturbs the kidney, and kidney disease may induce genital disease. Again, it may be observed that the peritoneum is very intimately associated with the genitals and kidney, and injury to the peritoneum may be followed by kidney disease. I have seen a uterus removed per vaginum and the patient die five or six days after from nephritis. The test-tube was two-thirds full of albumen on the application of heat. The genitals, peritoneum, and kidney are a triumvirate with close and intimate connections, both anatomically and physiologically. The disease of viscera which results from tumors, especially kidney disease, will demand more and more attention from gynecologists.

Women frequently die after laparatomy from nephritis, induced by the reflex irritation from the operation. The anesthetic may aid in producing the nephritis if it be ether. Chloroform affects the heart. In operating on any patient, the standard measure should be the amount of urea to the ounce of urine. There should not be less than six grains of urea to the ounce of urine. The patient should lie down for several days before the operation, so that the kidney would be well prepared for the shock.

In regard to *infection* causing death, it is so well recognized that little need be said. In the past eighteen months I have lost only one case from peritonitis after laparatomy. We have learned to keep out peritonitis by cleanliness—by heat and water.

I would also add that much manipulation in the peritoneum abrades the epithelium of the peritoneum, and leaves wounded surfaces, which allow infection to get in the system easily. But as we have no measure, constitutionally, of resistance, so we have no measure against the susceptibility of a person to peritonitis. However, these few desultory remarks may call attention to the subjects of anesthesia, kidney disease, reflexes, and infection following laparatomy. It is hoped that some of the points may lead others to investigate each or every individual factor.

34 Washington Street.

